# A breakthrough in Maxillary LeFort II fracture reconstruction: Case series of rhinoplasty using diced cartilage fascia graft simultaneously with ORIF

**DOI:** 10.1016/j.amsu.2021.102312

**Published:** 2021-05-04

**Authors:** Indri Lakhsmi Putri, Wilma Agustina

**Affiliations:** Department of Plastic Reconstructive and Aesthetic Surgery, Faculty of Medicine, Airlangga University, Surabaya, East Java, Indonesia

**Keywords:** Midface fracture, Le Fort II, Rhinoplasty, Diced cartilage fascia graft

## Abstract

Maxillary Le Fort II fracture reconstruction plays one of the challenging surgery in the field of maxillofacial trauma. The goal of treatment is reduction, reposition, fixation of fractures and restoration of occlusion. However, it is often not enough to bring back the appearance aesthetically. The challenge that we face today is that patients frequently complain about their nose postoperatively, hence, they believe that the deformity still remains. Secondary rhinoplasty post-trauma is often performed to overcome this deformity. We proposed direct rhinoplasty using diced cartilage fascia graft in Maxillary Le Fort II fracture reconstruction to provides better post-op aesthetic appearance.

Reporting three cases of Maxillary Le Fort II fractures. All patients had undergone open reduction and internal fixation combined with rhinoplasty using diced cartilage wrapped with fascia.

The graft provides a better nasal contour and shape, also camouflage irregularities. There was no clinical signs of graft absorption or infection. The patients were satisfied, and none of the patients complaint about their nose after surgery.

Rhinoplasty using diced cartilage fascia graft simultaneously with ORIF is a breakthrough in Maxillary Le Fort II reconstruction. It brings off the incorporation of aesthetic surgery concept into reconstruction, annihilating post-op complaint from patients and preventing secondary rhinoplasty due to previous trauma.

## Highlights

• Rhinoplasty using diced cartilage fascia graft simultaneously with ORIF is a breakthrough in Maxillary Le Fort II reconstruction.
• It brings off the incorporation of aesthetic surgery concept into reconstruction, annihilating post-op complaint from patients and preventing secondary rhinoplasty due to previous trauma.

## 1 Introduction

Rene LeFort in 1901 classified midface fractures as I, II, or III based on three great lines of weakness that correspond to the most common fracture sites [1]. It extends from or below nasofrontal suture through the frontal processes of maxilla, inferolaterally through lacrimal bones and inferior orbital floor. Furthermore, it transits the rim or near inferior orbital foramen, and inferiorly through anterior wall of maxillary sinus, it then travels below the zygoma, across pterygomaxillary fissure, and through the pterygoid plates, hence, it is aptly described as pyramidal fractures [2,3].

Maxilla Le Fort II fracture reconstruction consitute one of the challenging surgery in the field of maxillofacial trauma. Meanwhile, the nose is one of the most distinct and prominent features of the face [4]. It determines human races, and play essential roles in everyday life. When an individual has functional disorder or appears unhappy with the look on the nose, it compromise the quality of life. Moreover, the natural projection and the fragility of structures in this organ contribute to its susceptibility for injury. The nose also provides both aesthetic and structural support for midface and airway, hence, minor nasal traumas results in significant aesthetic and or functional defects [4]. Meanwhile, the LeFort fracturessurgical intervention is aimed at restoring occlusion, facial buttresses, as well as midface height, width, projection, and integrity of the nose and orbit [1,2]. However, it is often not enough to bring back the appearance aesthetically post reconstruction. The challenge that we face today is that patients regularly complaint about the fact that their nose is flat and wide after reconstruction, especially around the frontonasal area. Therefore, they believe that the deformity still remains, even after the open reduction and internal fixation (ORIF). Secondary rhinoplasty post-trauma is often performed to overcome this deformity.

Meanwhile, diced cartilage graft gained attention after publication by Erol with a technique termed Turkish delight, using surgicel wrapped diced cartilage grafts for rhinoplasty [5]. It has advantages of improving nasal contour, correcting nasal asymmetry, restoring shape by camouflage overlay, and its malleability through finger manipulation or molding up to 3 weeks postop to correct deviation, irregularities, or the width of a graft [5,6]. This was modified by Daniel and Calvert [7] using fascia wrapped diced cartilage grafts for rhinoplasty. They mention a significant foreign body reaction histologically and poorer graft survival if wrapped in surgicel compared to fascia.

Nowadays, the diced cartilage fascia graft technique is used in various related clinical conditions. This is performed using both primary and secondary rhinoplasty and serve as a reliable method for correcting significant problems resulting from previous rhinoplasty or post-trauma, where augmentation or contouring is necessary [8]. We proposed direct rhinoplasty using diced cartilage fascia graft in Maxillary Le Fort II fracture reconstruction to provides better post-op aesthetic appearance. Meanwhile, all cases were reported in congruent with the PROCESS requirements [9].

## 2 Case presentation

**Fig. 1:**
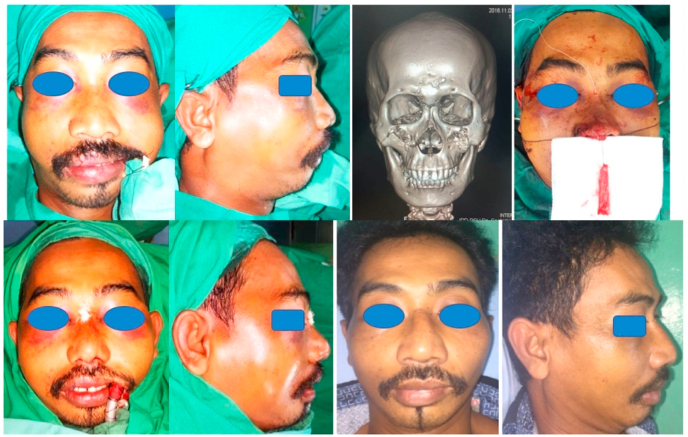
Above (Left to Right) Pre-op anterior view of the patient; Pre-op lateral view of the patient; Pre-op three-dimension CT scan of the patient; Pre-insertion of the diced cartilage fascia graft. Below (Left to right) Post-op anterior view of the patient; Post-op lateral view of the patient; One month post-op anterior view of the patient; One month post-op lateral view of the patient.

**Fig. 2:**
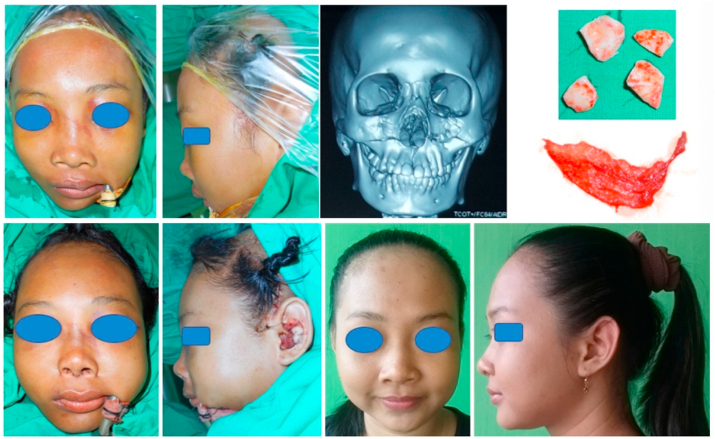
Above (Left to Right) Pre-op anterior view of the patient; Pre-op lateral view of the patient; Pre-op three-dimension CT scan of the patient; The conchal cartilage and superficial temporal fascia of the patient. Below (Left to right) Post-op anterior view of the patient; Post-op lateral view of the patient; Four years post-op anterior view of the patient; Four years post-op lateral view of the patient.

**Fig. 3:**
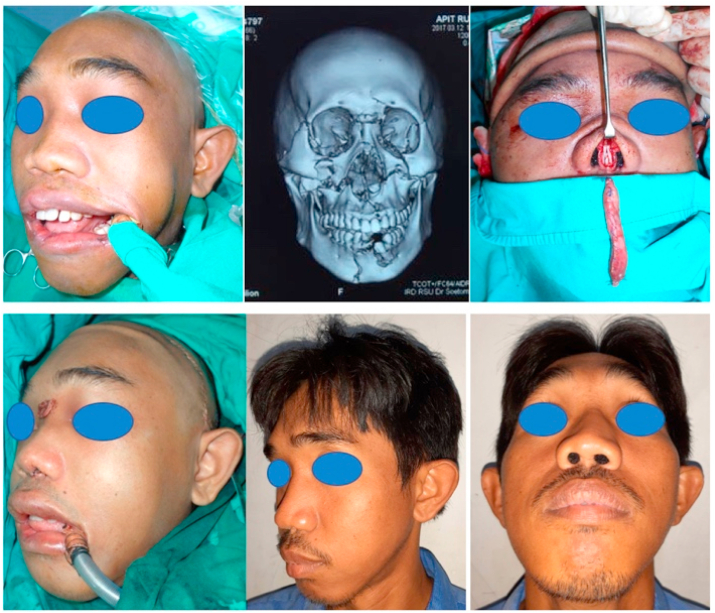
Above (Left to Right) Pre-op oblique view of the patient; Pre-op three-dimension CT scan of the patient; Pre-insertion of the diced cartilage fascia graft. Below (Left to right) Post-op oblique lateral view of the patient; Four years post-op oblique view of the patient; Four years post-op malar view of the patient.

We reported a retrospective, single centre and non-consecutive case series consisting three patients diagnosed with Maxillary Le Fort II fracture (see Fig. 1, Fig. 2, Fig. 3). All patient arrived at hospital urgently by ambulance. All surgery were performed by plastic surgeons with 4 years of experience. The patient were discharged 5 days post-op.

### 2.1 Case I

A 45 year old man was diagnosed Maxillary Le Fort II fracture due to a fall from motorcycle. There was a comminuted nasal fracture without complaint about difficulty of breathing.

An open reduction and internal fixation using miniplate was performed to treat the fracture using intra oral approach through upper gingivobuccal sulcus incision and subcilliary lower eyelid incision. Then, open rhinoplasty approach was performed. Cartilage graft was harvested from the 6th rib then evenly diced into small pieces and being inserted into 1 cc dysposable syringe. Fascia lata was harvested and being sutured in cylindrical form covering the syringe filled with diced cartilage, just before final sutures the diced cartilage was injected into the fascia, then final suture was performed sealing the fascia, forming a diced cartilage fascia graft with a cylindrical tube shape. Then, the diced cartilage fascia graft was inserted into the dorsal nasal skin and fixed using a non-absorbable suture in the frontonasal area. Periosteal and alar cinch suture also performed during soft tissue closure.

The nose is then taped and casted. Nasal packing were placed and remained in the nose for 10 days under oral amocixillin treatment.

### 2.2 Case II

A 16 year old woman was diagnosed Maxillary Le Fort I and II fracture due to a fall from motorcycle. There was comminuted nasal fracture without complaint about difficulty of breathing. An open reduction and internal fixation using miniplate was performed to treat the fracture using bicoronal approach, intra oral approach through upper gingivobuccal sulcus incisions and subcilliary lower eyelid incisions. Then, open rhinoplasty approach was performed. Cartilage graft was harvested from the conchal auricula then evenly diced into small pieces and being inserted into 1 cc dysposable syringe. Superficial temporal fascia was harvested and being sutured in cylindrical form covering the syringe filled with diced cartilage, just before final sutures the diced cartilage was injected into the fascia, then final suture was performed sealing the fascia, forming a diced cartilage fascia graft with a cylindrical tube shape. Then, the diced cartilage fascia graft was inserted into the dorsal nasal skin and fixed using a non-absorbable suture in the frontonasal area. Periosteal and alar cinch suture also performed during soft tissue closure.

The nose is then taped and casted. Nasal packing were placed and remained in the nose for 10 days under oral amocixillin treatment.

### 2.3 Case III

A 27 year old man was diagnosed Maxillary Le Fort I, II and III also mandible fractures due to a fall from motorcycle. There was comminuted nasorbitoethmoid and nasal septum fracture without complaint about difficulty of breathing. An open reduction and internal fixation using miniplate was performed to treat the fracture using bicoronal approach, intra oral approach through upper and lower gingivobuccal sulcus incisions also subcilliary lower eyelid incisions. Then, open rhinoplasty approach was performed. Cartilage graft was harvested from the 6th rib, a small piece of the cartilage graft was inserted into the septum and fixed with 5–0 non absorbable suture, the leftovers cartilage then evenly diced into small pieces and being inserted into 1 cc dysposable syringe. Superficial temporal fascia was harvested and being sutured in cylindrical form covering the syringe filled with diced cartilage, just before final sutures the diced cartilage was injected into the fascia, then final suture was performed sealing the fascia, forming a diced cartilage fascia graft with a cylindrical tube shape. Then, the diced cartilage fascia graft was inserted into the dorsal nasal skin and fixed using a non-absorbable suture in the frontonasal area. Periosteal and alar cinch suture also performed during soft tissue closure.

The nose is then taped and casted. Nasal packing were placed and remained in the nose for 10 days under oral amocixillin treatment.

## 3 Results

All patients were followed up until four years post-op in outpatient clinic and via online consultation. The dental occlusions were good and we found no clinical sign of infection or graft absorption. All of the patients were satisfied, no further complaint about nasal appearance, irregularities, contour or shape from the patients.

## 4 Discussion

The main goal of Maxillary Lefort II fractures treatment is to establish occlusion, restore the vertical and horizontal buttresses to re-establish the midface *structure and aesthetics* respectively, as well as the appearance in terms of facial integrity, projection, height and width [1,2]. Besides, other essential treatment considerations include early one-stage repair, precise reduction, wide exposure of all fractured segments, rigid fixation, simultaneous soft tissue repair(when necessary) as well as autogeneous bone grafts [1]. Meanwhile, sequencing Lefort fracture reconstruction is also important but somewhat controversial. In this study, the reconstruction involved of the following: placement of arch bars, exposure of all fractures site, reduction and reposition, placement into occlusion, fixation using miniplate, rhinoplasty using diced cartilage fascia graft, resuspension and soft tissue repair.

The two major facial buttresses of midface are vertical and horizontal buttresses. The vertical midface buttresses of the face are nasomaxillary, zygomaticomaxillary and pterygomaxillary. The horizontal midface buttresses of the face are frontal bar, orbital rims and maxillary alveolar. The treatment consisted of internal fixation with plates and screws of the buttresses, meanwhile, to withstand functional vertical bite loads on the buttresses, the plates are required to be of adequate size. Bone grafts are used when necessary to bridge bone defects in form of gaps greater than 5–10 mm [1,2].

In terms of surgical approaches of Maxillary Lefort II Fracture [1,2], the mainstay for exposure is intra oral approach through upper gingivobuccal sulcus incision, which allows excellent exposure of the medial and lateral vertical buttresses. Lower eyelid incisions, such as the subcilliary or trans conjuctival incisions, allow for exposure of horizontal buttress, the infraorbital rims. The frontozygomatic fracture which located in zygomaticomaxillary vertical buttress can be exposed through periorbital incision or lateral eyebrow incision. Furthermore, the coronal approach provides adequate exposure to fractures located in nasomaxillary vertical buttress when QRIF is indicated. Correction of traumatic and iatrogenic soft tissue injuries constitute the final component of the reconstruction, meanwhile, soft tissues are to be resuspended when the wide subperiosteal dissection is performed, to prevent midfacial tissues obligatory ptosis [1]. In our cases, we performed periosteal resuspension suture to prevent ptosis of midfacial tissues together with alar cinch resuspension suture to prevent alar widening post-op.

The fractures that appeared in Le Fort II fracture consists of the nasal part of the face. Besides, the unique anatomic features associated with the nose including aesthetic, structural, and functional necessitate a thorough understanding. Hence, restoring the integrity of the nose become one of the goals of surgical intervention of LeFort II fractures. This lead to the fact that patients may hope that their appearance will come back as it did before, or at least close to. In plastic surgery, rhinoplasty consitute one of the most challenging operations, it is usually frustrating for both surgeon and patient alike hence, the procedure is often regarded as a “continuing quest”. Functional rhinoplasty aside from correcting physical abnormalities, it can also correct the effects of physical trauma, restoring patient confidence with their appearance.

Autologous tissue rarely cause implant-related (exposure through the tip skin or vestibular mucosa) complications including infection, dorsal skin thinning and redness, as well as capsular contracture [6]. Solid autologous onlay grafts prepared from the costal cartilage for nasal augmentation are associated with high rates of revision due to several problems, including resorption, warping, and graft visibility in the long term [10]. Meanwhile, using camouflage overlay, cartilage grafts are widely utilized to improve contour of the nose [5]. Nowadays, diced cartilage graft is considered as the gold standard for dorsal augmentation due to its versatility with the use of autologous tissues [11,12]. Besides, It is a useful stand-alone technique or in conjunction with other procedures, and is probably the best filler available to camouflage various forms of nasal defects [8,13]. Advantages of diced cartilage grafts compared to the range of graft material used in rhinoplasty surgery are [5,11,[13], [14], [15]];

1. Utilizes autogenous material, hence, rejection is not an issue
2. Survives as living tissue with optimal biocompatibility.
3. Easily prepared.
4. All types of cartilage are suitable
5. Used in various forms, freely or along with fascia or surgicel
6. Designed into a wide range of shapes to fit various recipient sites.
7. Camouflages various forms of nasal defects
8. It is malleable and offers unparalleled flexibility, which allows for fine adjustments both intraoperatively and up to 3 weeks postoperatively.
9. The entire process is relatively simple, not technically demanding, easy to learn even to inexperienced surgeons and can be performed quickly.
10. There is no possibility of warping.
11. It is low risk of infection or extrusion.
12. Less visibility thus more natural result
13. Revision is easily corrected by shaving with a knife blade or using a percutaneous no. 16 needle.

The only inherent disadvantages of diced cartilage grafts is not structural grafts, and from technical perspective are overcorrection, visibility, junctional step-offs [13]. Meanwhile, resorption is controversial based on the reports from various authors. Daniel [13] reported no evidence of absorption in cases performed with a mean follow-up of 3 years. Furthermore, Suh [6] suggests to minimize dead space by finely dicing the cartilage and packing the particles into the fascia tube to prevent subsequent reduction.

However, in recent decades, an interest in using diced cartilage covered with fascia for primary and secondary rhinoplasty procedures has resurfaced, it doesn't induce foreign body reaction histologically with better graft survival. The surgeon believed that doing diced cartilage fascia graft while performing ORIF in Maxillary Lefort II Fracture cases will restore integrity of the nose, annihilating complaint about post op nasal aesthetic appearance. The released soft tissues must not only be resuspended, but also augmented to prevent ptosis after Maxillary Lefort II Fracture reconstruction.

The advantages of augmenting the nose with diced cartilage fascia graft while performing ORIF in Maxillary Lefort II Fracture reconstruction are provide best camouflage overlay, autogenous origin thereby no rejection and highly resistant of infection, easy and simple procedures and can be manipulate with palpation or molding up to 3 weeks post op. However, it has disadvantages too, such as adding more time in surgery (1 h more), adding more surgery site, donor's site morbidity include more pain and another scar for the patient. Regarding it could annihilate post op complaints about nasal aesthetic appearance from the patient, which could lead to secondary surgery, we believed that doing rhinoplasty using diced cartilage fascia graft together with ORIF will be a better solution for both patient and surgeon, preventing secondary rhinoplasty due to previous trauma. It is not only considering reduction, reposition, fixation of fractures and restoration of dental occlusion but also the final aesthetic appearance of the patients.

## 5 Conclusion

Maxillary Le Fort II reconstruction using ORIF combined with rhinoplasty using diced cartilage fascia graft is a breakthrough in Maxillary Le Fort II fracture management. The procedure consist of reduction, reposition, fixation of fractures, restoration of dental occlusion, resuspension and augmentation of soft tissues. It is safe, easy to use and brings off the incorporation between aesthetic surgery concept with reconstruction. It will restore integrity of the nose, annihilating post op complaint from patients and preventing secondary rhinoplasty due to previous trauma. This may be a very promising procedure for better future clinical practice of Maxillary Le Fort II fracture.

## Ethical approval

It is declared in the written informed consent that patient data will be used for educational and research purposed. Our institution does not provide an ethical approval in the form of case series.

## Sources of funding

No funding source to declare.

## Author contributions

Indri Lakhsmi Putri: Study concept, resources, data analysis, writing-review & editing, final approval of the version to be submitted.

Wilma Agustina: Data collection, data analysis, writing-original draft.

## Research registration number

Researchregistry6652.

## Guarantor

Indri Lakhsmi Putri.

## Consent

For publication of this case report, an informed consent was obtained from the patient with accompanying images. A copy is available with the Editor-in-Chief for review on request.

## Provenance and peer review

Not commissioned, externally peer-reviewed.

## Declaration of competing interest

No potential conflict of interest.
